# The long noncoding RNA of RMRP is downregulated by PERK, which induces apoptosis in hepatocellular carcinoma cells

**DOI:** 10.1038/s41598-021-86592-6

**Published:** 2021-04-12

**Authors:** Atsushi Yukimoto, Takao Watanabe, Kotaro Sunago, Yoshiko Nakamura, Takaaki Tanaka, Yohei Koizumi, Osamu Yoshida, Yoshio Tokumoto, Masashi Hirooka, Masanori Abe, Yoichi Hiasa

**Affiliations:** Department of Gastroenterology and Metabology, Ehime University Graduate School of Medicine, Shitsukawa 454, Toon, Ehime 791-0295 Japan

**Keywords:** Liver diseases, Hepatocellular carcinoma

## Abstract

Endoplasmic reticulum (ER) stress plays an important role in hepatocyte degeneration, especially in patients with chronic liver injury. Protein kinase R-like endoplasmic reticulum kinase (PERK) is a key molecule in ER stress. PERK may contribute to apoptotic cell death in HCC, however the details of the mechanism are not clear. In this study, we identified PERK-associated molecules using transcriptome analysis. We modulated PERK expression using a plasmid, tunicamycin and siRNA against PERK, and then confirmed the target gene expression with real-time PCR and Northern blotting. We further analyzed the apoptotic function. Transcriptome analysis revealed that expression of the RNA component of mitochondrial RNA processing endoribonuclease (RMRP), which is a long noncoding RNA, was strongly correlated with the function of PERK. The expression of RMRP was correlated with the expression of PERK in experiments with the siRNA and PERK plasmid in both HCC cell lines and human HCC tissue. Furthermore, RMRP downregulation induced apoptotic cell death. RMRP is downregulated by PERK, which induces apoptosis in HCC. RMRP could be a new therapeutic target to regulate HCC in patients with chronic liver diseases associated with ER stress.

## Introduction

Hepatocellular carcinoma (HCC) is the third most common cause of cancer death in the world^1^. Molecular targeted drugs such as tyrosine kinase inhibitors have therapeutic effects in HCC patients^2^. However, treating patients with lower hepatic function reserve, and treatment effects are limited. New therapeutic targets for treating HCC are therefore required.

ER stress is triggered by inhibition of protein glycosylation, disruptions in calcium ion homeostasis, and hypoxia^3^ and induces the unfolded protein response (UPR). UPR is a phenomenon by which cells escape from damage and is involved in various diseases, such as cardiovascular disease^4^, Alzheimer disease^5^, and cancer^6–8^.

In hepatology, ER stress is a risk factor for chronic liver injury, especially in patients with NASH. In NASH model mice, ER stress in hepatocytes is increased by a high fat diet and leads to steatohepatitis^9^. A previous study revealed that ER stress plays an important role in the pathogenesis of NASH^10^. In HCC, induction of ER stress may lead to cell death^11^. On the other hand, after adaptation to ER stress, the tumor size increases^12,13^. ER stress is strongly related to cell survival and death in chronic liver injury and carcinogenesis.

PERK, inositol requiring enzyme 1, and activating transcription factor 6 are key regulators of ER stress. Among these molecules, we focused on PERK. PERK and PKR, which are both kinases that activate eukaryotic translation initiation factor 2-alpha (eIF2α), catalyze an early regulated step of protein synthesis initiation. PKR is a soluble molecule that exists in the cytosol, whereas PERK is expressed in the ER membrane. The factors that activate these two molecules are not the same. For example, PKR expression is stimulated by viral infections such as hepatitis C, whereas PERK expression is stimulated by ER stress^14^. In a previous study, we reported that PKR is overexpressed and activated in HCC compared with surrounding non-HCC tissues^15^. PKR is also related to cancer proliferation^16^. PKR affects hepatic satellite cells and promotes proliferation of HCC^17^. These results indicate that PKR plays a role in cancer proliferation. PERK has high amino acid homology with PKR, suggesting a relationship between PERK and HCC.

Regarding the relationship between PERK expression and malignant diseases, PERK is an anti-proliferative molecule that retains a restriction point. Ectopic expression of PERK in tumors suppresses the growth of cancer cells in vivo^18^, showing that PERK has tumor-suppressor function. However, adaption to ER stress induces upregulation of molecules that are needed for survival of cancer cells including HCC^13,19^. To clarify the mechanism of ER stress in HCC, we need to identify another key downstream molecule that regulates HCC proliferation. We first identified a molecule known as RNA component of mitochondrial RNA processing endoribonuclease (RMRP), which is strongly linked to PERK. We evaluated the expression of RMRP in HCC and whether this molecule has critical effects for patients with HCC.

## Results

### Knock down of PERK expression with siRNA leads to an increase in RMRP

**Figure 1:**
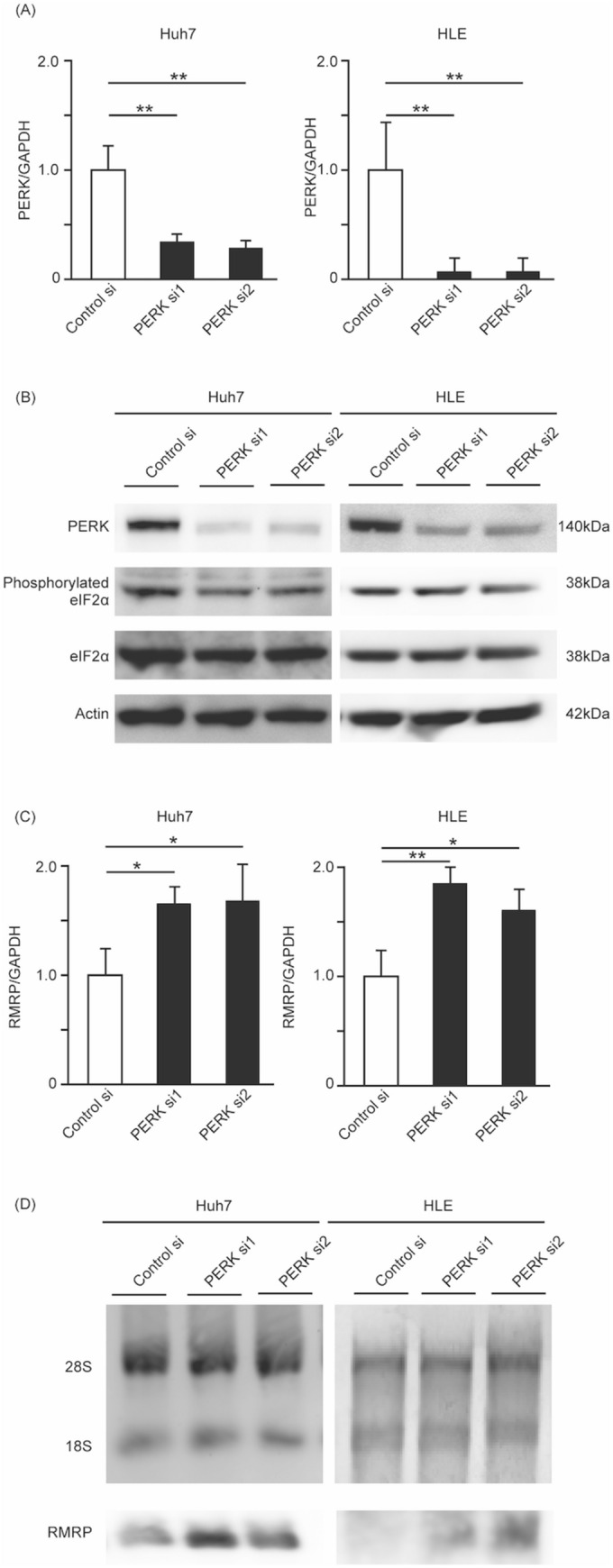
Silencing PERK regulated RMRP expression. Huh7 and HLE cells were seeded in a 6-well flat-bottomed plate and transfected with control siRNA, PERK siRNA1, or PERK siRNA2. 24 h after treatment, RNAs and proteins were extracted and analyzed. mRNA expression of PERK was measured with RT-PCR. PERK expression was markedly suppressed by PERK siRNA. Means ± SEM for six replicates are shown. **p < 0.01 compared to control siRNA group with the Student’s t-test (**A**). Levels of PERK, eIF2α, and phosphorylated eIF2α were determined with Western blotting. The anti-PERK antibody, anti-eIF2α antibody and anti-phospho eIF2α antibody using in this figure were product number anti-PERK (product number: 3192), anti-eIF2α (5324), anti-phospho eIF2α (3398). PERK and phosphorylated eIF2α were suppressed by PERK siRNA. eIF2α expression was not changed between control siRNA group and PERK siRNA group. The original gel images of Western blotting are shown in Supplemental Fig. S1 (**B**). RNA expression of RMRP was measured with RT-PCR analysis. PERK downregulation led to increase RMRP expression. Means ± SEM for six replicates are shown. *p < 0.05 and **p < 0.01 compared to the control siRNA group with the Student’s t-test (**C**). Levels of RMRP were determined with Northern blotting. 28S and 18S are shown as control bands. The original gel images of Northern blotting are shown in Supplemental Fig. S2 (**D**).

To investigate PERK-related genes, we transfected PERK siRNA into Huh7 and HLE cells. We confirmed that PERK mRNA expression and protein translation were knocked down, using RT-PCR (Fig. 1A) and Western blotting (Fig. 1B). Expression of phosphorylated eIF2α was also decreased. Thus, PERK signaling was inactivated by siRNA transfection in Huh7 and HLE cells.

We then used next generation sequencing to reveal the expression change between PERK siRNA-transfected cells and control siRNA-transfected cells. We identified RMRP as being remarkably increased in PERK siRNA-transfected cells (Supplemental Table 1). To ensure that downregulated PERK expression leads to RMRP upregulation, we use real-time RT-PCR and Northern blotting. Two kinds of PERK siRNA were used to inhibit PERK expression, and decreased PERK expression led to increased RMRP (Fig. 1C). We obtained the same results with Northern blotting (Fig. 1D), clearly indicating a relationship between PERK and RMRP expression.

### PERK overexpression decreases RMRP

**Figure 2:**
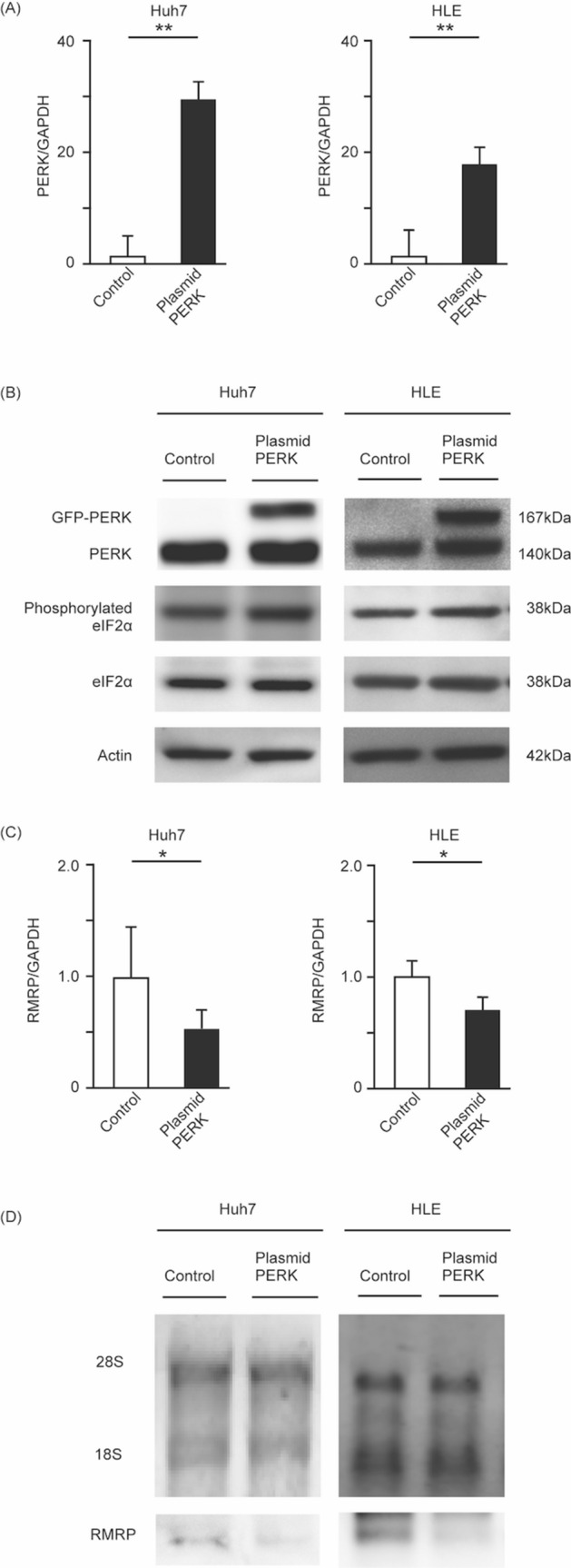
PERK overexpression leads to RMRP downregulation. Huh7 and HLE cells were seeded in a 6-well flat-bottomed plate and transfected with control plasmid or PERK overexpression plasmid. 24 h after treatment, RNAs and proteins were extracted and analyzed. Fold changes in PERK mRNA expression were measured with RT-PCR. PERK expression was increased by PERK overexpression plasmid. Means ± SEM for six replicates are shown. **p < 0.01 compared to the DMSO group with the Student’s t-test (**A**). Levels of PERK, eIF2α, and phosphorylated eIF2α were determined with Western blotting. Phosphorylated eIF2α was increased by PERK overexpression plasmid. eIF2α expression was not changed between control group and PERK overexpression group. The original gel images of Western blotting are shown in Supplemental Fig. S3 (**B**). Expression of RMRP was measured with RT-PCR. RMRP expression was decreased by PERK overexpression plasmid. Means ± SEM for six replicates are shown. *p < 0.05 compared to the control plasmid group with the Student’s t-test (**C**). Levels of RMRP were determined with Northern blotting. 28S and 18S are shown as control bands. The original gel images of Northern blotting are shown in Supplemental Fig. S4 (**D**).

We next conducted PERK overexpression experiments using a PERK expression plasmid. We transfected HCC cell lines with PERK expression or control plasmids, and then extracted RNA and protein. We verified that the RNA level of PERK was increased in PERK-overexpressing cells compared to the control group (Fig. 2A). Western blotting analysis showed that exogenous PERK expression appeared at 24 kDa, which was above the endogenous PERK protein band because of the green fluorescent protein sequence (Fig. 2B). Phosphorylated eIF2α expression was also increased, confirming that the PERK expression plasmid was functional. In PERK-overexpressing cells, the expression of RMRP was downregulated, as seen with RT-PCR (Fig. 2C) and Northern blotting (Fig. 2D).

### ER stress also decreases RMRP expression

**Figure 3:**
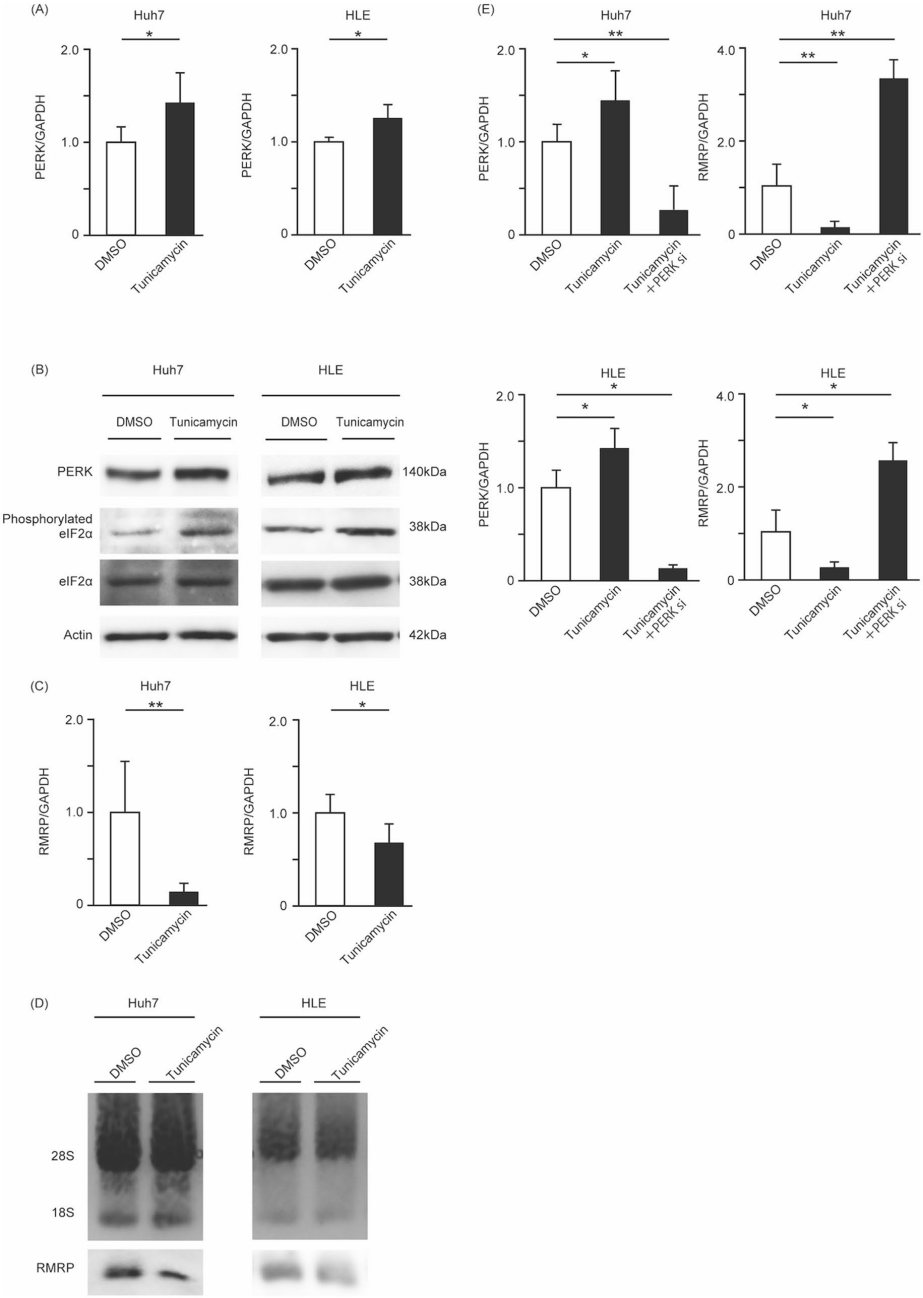
ER stress induced by tunicamycin leads to RMRP downregulation. After incubation in a six-well flat-bottom plate, Huh7 and HLE cells were treated with DMSO or 1 μg/mL tunicamycin for 24 h. After treatment, RNAs and proteins were extracted and analyzed. PERK mRNA expression was measured with RT-PCR. PERK expression was increased in the tunicamycin group. Means ± SEM for six replicates are shown. *p < 0.05 compared to the DMSO group with the Student’s t-test (**A**). Levels of PERK, eIF2α, and phosphorylated eIF2α were determined with Western blotting. PERK and phosphorylated eIF2α were increased by tunicamycin. eIF2α expression is not changed between control group and tunicamycin group. The original gel images of Western blotting are shown in Supplemental Fig. S5(**B**). Expression of RMRP RNA was measured with RT-PCR. RMRP expression was decreased by tunicamycin treatment. Means ± SEM for six replicates are shown. **p < 0.01 and *p < 0.05 compared to the DMSO group with the Student’s t-test (**C**). Levels of RMRP were determined with Northern blotting. 28S and 18S are shown as control bands. The original gel images of Northern blotting are shown in Supplemental Fig. S6(**D**). Fold change in PERK and RMRP was measured with RT-PCR compared to the DMSO group. PERK expression was increased by tunicamycin and decreased by tunicamycin + PERK siRNA. RMRP expression was decreased by tunicamycin treatment. In tunicamycin + PERK siRNA group, the RMRP expression level was increased significantly. Means ± SEM for six replicates are shown. **p < 0.01 and *p < 0.05 compared to the control DMSO group, as determined by one-way analysis of variance (**E**).

Because PERK is an ER stress molecule, we investigated the influence of ER stress on RMRP by adding tunicamycin dissolved in DMSO to Huh7 and HLE cells. As seen with RT-PCR, in the tunicamycin treatment group, PERK mRNA expression was increased (Fig. 3A). We also confirmed the upregulation of PERK protein with Western blotting (Fig. 3B). Increased expression of phosphorylated eIF2α indicated that the upregulated PERK was functional. In these cells, RMRP was decreased as seen with RT-PCR (Fig. 3C) and Northern blotting (Fig. 3D). Then, we transfected cells with PERK siRNA after treatment with tunicamycin. As a result, the RMRP expression level was increased significantly (Fig. 3E). These results indicate that PERK and RMRP function inversely in the same pathway that is involved in ER stress.

### RMRP expression influences apoptosis

**Figure 4:**
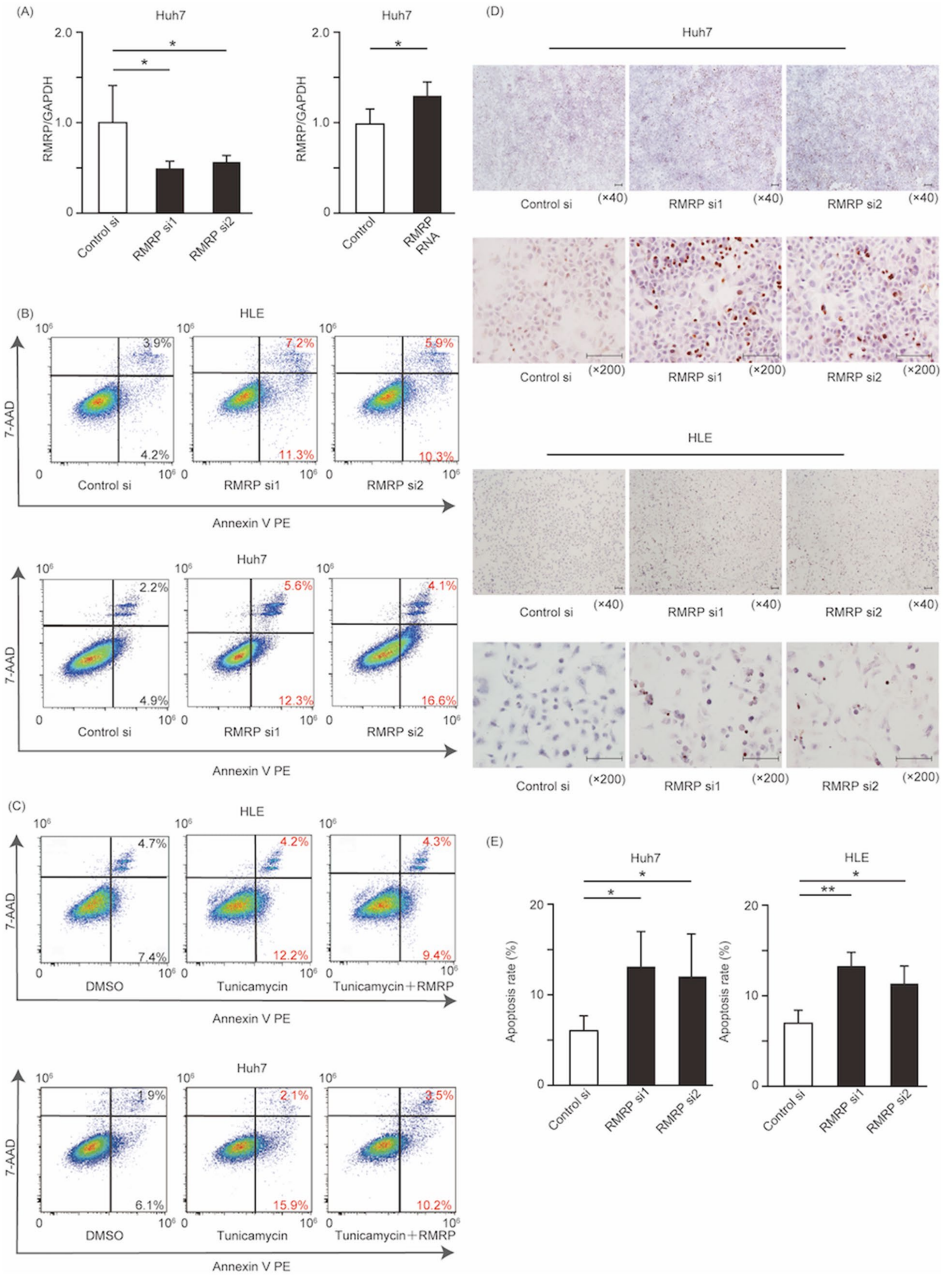
Silencing of RMRP leads to apoptosis in Huh7 and HLE cells. Huh7 and HLE cells were transfected with control siRNA, RMRP siRNA, or RMRP RNA for 24 h. Fold changes in RMRP were measured with RT-PCR. Means ± SEM for six replicates are shown. *p < 0.05 compared to the control siRNA group with the Student’s t-test (**A**). Apoptotic effect of RMRP knockdown. Apoptotic cells were positive for annexin V, and necrotic cells were positive for 7-amino-actinomycin D (**B**). Control siRNA and RMRP RNA were transfected at 50 pmol/L. Huh7 and HLE cells were treated with DMSO, tunicamycin, or tunicamycin + RMRP RNA (**C**). TUNEL staining of Huh7 and HLE cells. Cells transfected with control siRNA, RMRP siRNA1, and RMRP siRNA2 are shown. Scale bar represents 100 μm (**D**). Rates of apoptotic cells according to TUNEL staining. Apoptotic cells were evaluated with ImageJ software (National Institutes of Health, Bethesda, MD, USA). Means ± SEM for five replicates are shown. **p < 0.01 and *p < 0.05 compared to the control siRNA group with the Student’s t-test (**E**).

We next investigated the function of RMRP. To knock down RMRP expression, we used two different RMRP siRNAs. To confirm whether synthesized RMRP RNA was transfected into cells actually, we analyzed RMRP expression levels in RMRP RNA-transfected cells and control siRNA-transfected cells with real-time RT-PCR. RMRP RNA-transfected cells showed clearly increased RMRP expression (Fig. 4A).

As seen with flow cytometry analysis, annexin V-positive cells were defined as apoptotic cells. The percentages of apoptotic cells were: control siRNA, 8.1%; RMRP siRNA1, 18.5%; and RMRP siRNA2, 16.2% in HLE cells. In Huh7 cells, the percentages were: control siRNA, 7.1%; RMRP siRNA1, 17.9%; and RMRP siRNA2, 20.7%. The rates of apoptosis clearly increased after 24-h tunicamycin treatment. However, after simultaneous RMRP RNA transfection, the rates of apoptosis decreased compared with tunicamycin treatment alone (Fig. 4B, C).

TUNEL analysis also revealed that in the RMRP siRNA1 and RMRP siRNA2 groups, apoptotic cells were increased compared with the control siRNA group (Fig. 4D). The rate of apoptotic cells is also shown in Fig. 4E.

### RMRP downregulation increases miR-206 and activates the apoptosis pathway

**Figure 5:**
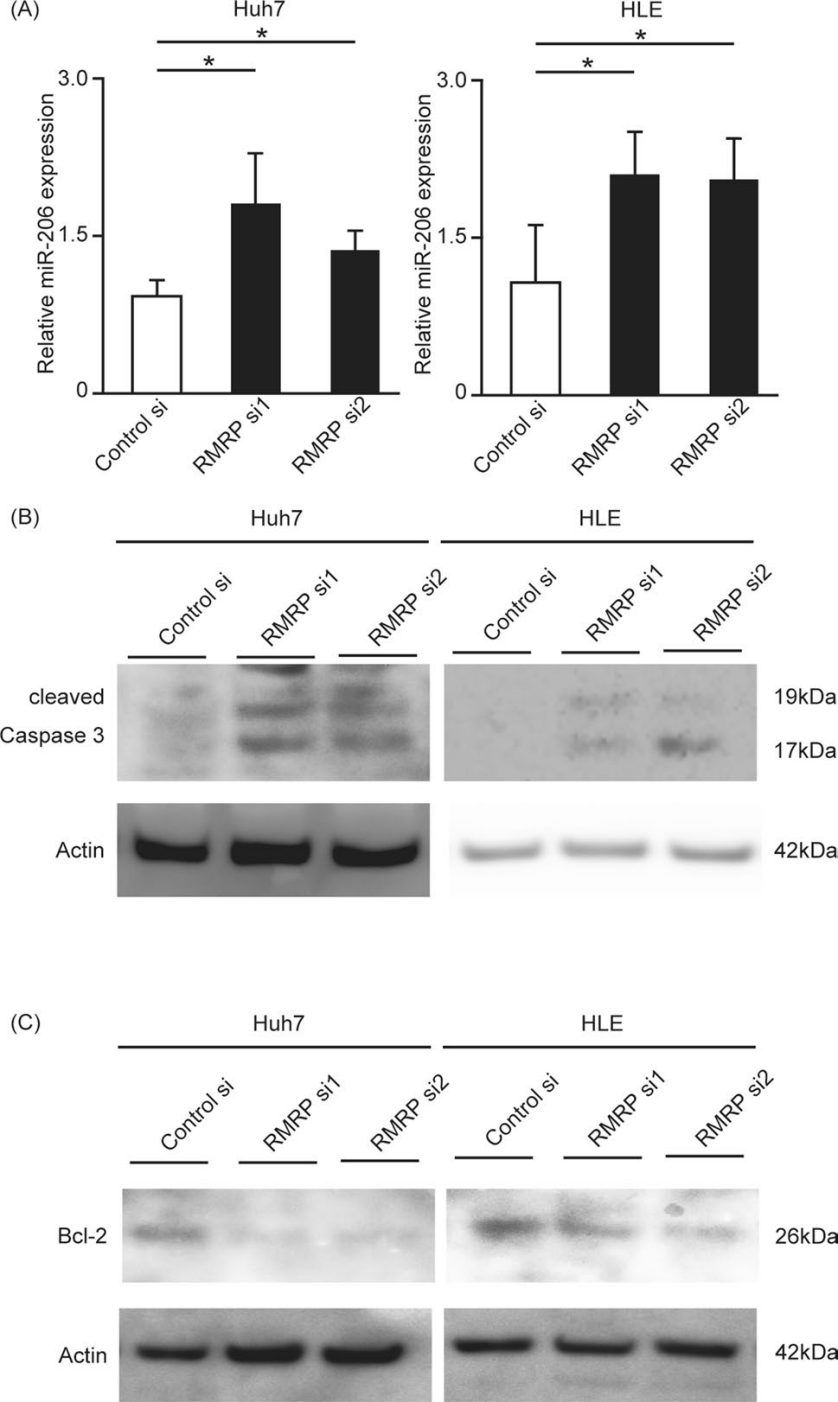
Silencing RMRP leads to an increase in miR-206 and changes cleaved caspase 3 and Bcl-2 expression. Huh7 cells were transfected with control siRNA or RMRP siRNA for 24 h. After transfection, miRNA was extracted, and miR-206 was measured with RT-PCR. miR-206 was increased by RMRP knockdown. Means ± SEM for six replicates are shown. *p < 0.05 compared to the control group by Student’s t-test (**A**). Levels of cleaved caspase 3 (**B**) and Bcl-2 (**C**) were determined with Western blotting. Cleaved caspase 3 was increased and Bcl-2 was decreased by RMRP siRNA. The original gel images of Western blotting are shown in Supplemental Fig. S8.

We showed that RMRP knockdown induces apoptosis. To investigate how RMRP induced apoptosis, we analyzed miR-206 expression and caspases. RMRP downregulation increased miR-206 and inhibited Bcl-2, and consequently induced cleaved caspase 3 (Fig. 5). RMRP silencing also led to activation of caspase 9 and caspase 3/7, but not caspase 8 (Fig. S7). These results indicated that the PERK-RMRP apoptotic pathway includes miR-206 and Bcl-2, and moreover, suggested that apoptosis due to the PERK-RMRP axis is correlated not with the extrinsic pathway but with the intrinsic pathway (mitochondrial apoptosis).

**Table 1:** Baseline characteristics of 32 patients with HCC.

**General parameters (n = 32)**	
Age (years); median (range)	70 (63–76)
Sex, male/female (n)	25/7
Etiology, HCV/HBV/NBNC (n)	12/9/11
Aspartate aminotransferase (IU/L); median (range)	46 (31–69)
Alanine aminotransferase (IU/L); median (range)	44 (25–68)
Platelets (10 × 10^4^ cells/μL); median (range)	14.9 (10.2–21.0)
Albumin (g/dL); median (range)	3.8 (3.4–4.0)
Bilirubin (mg/dL); median (range)	0.7 (0.6–1.0)
Prothrombin Time (%); median (range)	86.4 (78.9–97.7)
Alpha-fetoprotein (ng/mL); median (range)	23 (6–131)
DCP (mAU/mL); median (range)	378 (64–2171)
Child Pugh class A/B (n)	30/2
Tumor size (cm), median (range)	3.6 (2.5–6.8)
Tumor differentiation (well/moderate/poor)	8/21/3
Tumor multiplicity (solitary/multiple)	27/5

### The relationship between PERK and RMRP in clinical specimens

**Figure 6:**
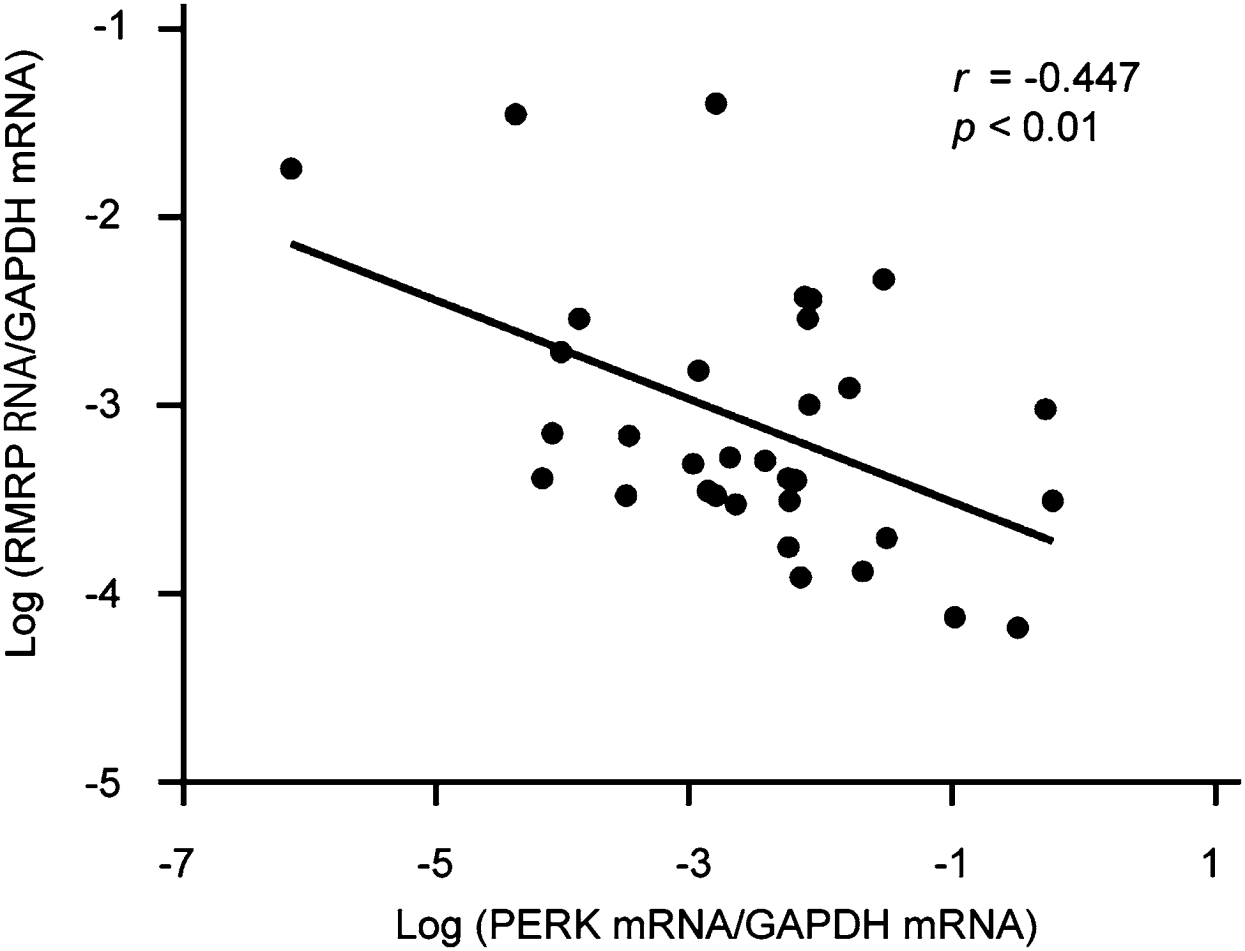
Expression of PERK and RMRP in human HCC tissues. RNA expression of PERK and RMRP was analyzed and compared, and the correlation coefficient was calculated by Pearson’s correlation coefficient. We found a negative correlation between PERK and RMRP expression in HCC specimens with real-time RT-PCR.

To investigate the clinical relationship between PERK and RMRP, we used HCC tissues collected during surgery. The clinical features of HCC patients are shown in Table 1. We found no difference in PERK expression according to the etiology of liver disease. We calculated the expression level of PERK RNA/GAPDH and RMRP RNA/GAPDH and found a negative correlation between PERK and RMRP expression in HCC specimens as seen with real-time RT-PCR (r =  −0.44, p < 0.01) (Fig. 6). These results strongly support the hypothesis that RMRP is a PERK downstream molecule and that this pathway mediates apoptosis induced by ER stress.

## Discussion

The results of the present study revealed that inactivating the PERK pathway induced RMRP upregulation. We activated PERK by using overexpression plasmids and tunicamycin. As a result, ER stress led to a decrease in RMRP expression. PERK and RMRP were also inversely expressed in clinical specimens. We found that RMRP was related to apoptosis, and that RMRP downregulation led to apoptosis in HCC cell lines.

The functions of long noncoding RNAs (lncRNAs) are still unclear. LncRNAs are defined as RNAs that are longer than 200 bases and that are non-protein-encoding. RMRP is a lncRNA that consists of 267 nucleotides and is unique because it is transcribed by RNA polymerase III instead of RNA polymerase II^20^. RMRP itself works as an endogenous siRNA with RNA-dependent RNA polymerase activity. RMRP is also an endonuclease that cleaves mitochondrial RNA^21^. In addition, RMRP is associated with hTERT^22^, and its RNA-dependent RNA polymerase activity produces double-stranded RNAs that can be processed into siRNA.

Mutations in the gene encoding RMRP are responsible for cartilage-hair hypoplasia, which is characterized by short stature, sparse hair, immune abnormalities, and a high incidence of malignancies^23^. In several cancers, RMRP expression is increased, and RMRP downregulation leads to cell cycle arrest and apoptosis. For example, in multiple lymphoma, c-Myc promotes the transcription of RMRP^24^, whereas in colon cancer, β-catenin enhances RMRP expression^25^. On the other hand, RMRP is decreased in gastric cancer tissues and in dysplasia tissues compared to control tissues^26^. The reason why RMRP is low in gastric cancer may be that ER stress affects RMRP expression via PERK. RMRP siRNA and RMRP overexpression have previously been used to change the expression of RMRP. However, how RMRP actually influences cancer progression is unclear, because examining RMRP plasmids that accurately transcribe RMRP RNA is difficult^27^. Therefore, in this study, we used RMRP RNA and confirmed its expression with real-time RT-PCR (Fig. 4A).

Previous studies have reported the downstream molecules of RMRP. RMRP is not translated into protein, but it influences miRNA; it stimulates miR-613^28^ and miR-206/TACR1 in HCC^29^. In several cancers, miR-206 is known as an apoptosis-related molecule^30,31^. Downregulated RMRP in our study increased miR-206, which targets Bcl-2 and consequently increases cleaved caspase 3 (Fig. 5), resulting in apoptosis. In contrast to downstream, molecules that are upstream of RMRP have remained unidentified. In this study, we revealed that PERK, an ER transmembrane protein, is an important upstream molecule of RMRP, which in turn mediates the apoptosis pathway.

Apoptosis is an important mechanism for suppressing cancer progression. Apoptosis is induced by ER stress via UPR signaling when the stress is excessive and brief^32^. Brief exposure to ER stress causes apoptosis of cancer cells^33^. ER stress activates PERK, and PERK inhibits the proliferative effects on HCC. PERK arrests the cell cycle and initiates apoptosis^34^. In this study, we used tunicamycin and PERK overexpression to activate transient ER stress, which induced apoptosis. Tunicamycin is useful as an ER stress inducer, because it inhibits GlcNac phosphotransferase to cause abnormal protein accumulation^35^. Tunicamycin also activates mTORC1 and induces apoptosis^36^. However, tunicamycin has not been used for cancer treatment because of its cytotoxicity. An RMRP-specific inhibitor could possibly be administered safely, and could be effective against HCC. The effect of tunicamycin, siRNA, and overexpressing plasmids is temporary, and thus, we need to confirm the continuous effects by downregulating RMRP in HCC in future work.

CHOP is a key molecule in the PERK apoptosis pathway^37,38^. CHOP, which forms a heterodimer with CCAAT/enhancer-binding protein or liver activator protein^39^, induces death by promoting protein synthesis and oxidation in the stressed ER^40,41^. CHOP then releases calcium from the ER into the cytoplasm to trigger apoptosis. We transfected HCC cells with RMRP siRNA but CHOP did not appear to show different expression in the control siRNA group compared to the RMRP siRNA group as seen with Western blotting (Fig. S9). We therefore suggest that RMRP is not upstream of the CHOP pathway. On the other hand, miR-206 is downstream of RMRP. In HCC, ER stress activates PERK, downregulates RMRP, and influences miR-206. This pathway inhibits Bcl-2 and activates caspase 9 and caspase 3, and thus, the mitochondrial apoptosis pathway is activated. As a result, the cells undergo apoptosis. We think that this process may complement previously known pathways.

The molecules that influence PERK activity have been described^42,43^. However, administration of these molecules is expected to have adverse effects, and thus, could not be applied in a clinical setting^44^. On the other hand, RMRP is further downstream of PERK, and no proteins are produced from RMRP because it is a non-coding RNA. Thus, an RMRP targeting strategy would be a novel therapeutic strategy that may not have serious adverse events. Inhibiting RMRP may be beneficial for suppression of HCC growth by inducing apoptosis of HCC cells. However, to obtain sufficient effects of lncRNA, we need to produce or identify a suitable delivery system for HCC cells. As a next step, we need to use a delivery system and animal models, and then confirm the anti-tumor effect of antisense RMRP in HCC.

In conclusion, we revealed a relationship between PERK and RMRP in vitro and in vivo. RMRP is downregulated by PERK and induces apoptosis in HCC cells. RMRP could be a new therapeutic target to regulate HCC in patients with chronic liver diseases associated with ER stress.

## Materials and methods

### Cell culture

We used the HCC cell lines, Huh7 and HLE (Japanese Collection of Research Bioresources, Osaka, Japan). Huh 7 and HLE cells were cultured in Dulbecco’s modified Eagle’s medium (DMEM) (Thermo Fisher Scientific, Waltham, MA) supplemented with 10% fetal bovine serum (Thermo Fisher Scientific) and 1% penicillin. Cells were grown in a humidified atmosphere of 5% CO_2_ and 95% air at 37 °C. The medium was replaced three times per week.

### RNA interference and tunicamycin assay

We used PERK specific siRNA1 (GAA GCU ACA UUG UCU AUU U, nt. 2424–2442), siRNA2 (UAG CAA AUC UUC UUC UGA A, nt. 2407–2426), and control siRNA (Dharmacon, Cambridge, UK). RMRP-specific siRNA1 (CCU AGG CUA CAC ACU GAG GAC UTT, nt. 22–44) and siRNA2 (GCC UGU AUC CUA GGC UAC ATT, nt. 14–33) were also obtained from Dharmacon. Huh7 and HLE cells were transfected with 50 pmol/L siRNA using RNAiMAX (Thermo Fisher Scientific). Twenty-four hours after transfection, RNA and protein were extracted from these cells. For the ER stress-induced assay, 1 μg/mL tunicamycin (Cayman Chemical Company, Ann Arbor, MI) was added to the cell culture, and the control group was treated with dimethyl sulfoxide (DMSO). After 24 h, RNA and protein were extracted from the cells.

### PERK plasmids

Plasmids encoding PERK were purchased from Origene (Rockville, MD) (RG214993). pCMV6 was used as a control plasmid. Plasmid (1 μg/mL) was transfected into Huh7 and HLE cells at 70% confluence using Lipofectamine 2000 (Thermo Fisher Scientific). After 24 h, RNA and protein were extracted from the cells.

### RNA extraction, cDNA synthesis, and real-time RT-PCR

From Huh7 cells, HLE cells, and clinical specimens, total RNA was extracted with an RNeasy Plus Mini Kit (Qiagen, Venro, Netherlands). Extracted RNA was reverse-transcribed using TaqMan Reverse Transcription Reagents (Thermo Fisher Scientific). For PCR amplification, we used a PERK forward primer (5′-CCAGCCTTAGCAAACCAGA-3′) and reverse primer (5′-TCTTGGTCCCACTFGGAAGAG-3′), total RMRP forward primer (5′-TGCTGAAGGCCTGTATCCT-3′) and reverse primer (5′-TGAGAATGAGCCCCGTGT-3′) and GAPDH primers (Hs.544577) (Thermo Fisher Scientific). Expression of each gene was normalized to GAPDH mRNA and is represented as fold changes compared to the control group. For the analysis of the relationship between PERK and RMRP, we used a Light Cycler LC480 (Roche, Rotkreuz, Switzerland). For PCR detection of miR-206 expression, we purchased supplies from Thermo Fisher Scientific, and U6 was used as an endogenous control. Analysis of the threshold cycles generated by qPCR was performed using the 2^−ΔΔCt^ method.

### Next generation sequencing

We prepared Huh7 cells and transfected them with control siRNA or PERK siRNA (n = 6). We used an Agilent 2200 TapeStation (Agilent Technologies, Santa Clara, CA) for quality control. Library construction and sequencing with the Illumina HiSeq 2500 were outsourced to Eurofins Genomics K. K. (Tokyo, Japan). Fragmented mRNA was reverse transcribed to cDNA. cDNA libraries were prepared following adaptor ligation, size selection (200-bp inserts), and amplification of cDNA. Libraries were used for paired-end 2 × 100 bp sequencing (HiSeq 2500) with v4 chemistry (Illumina, San Diego, CA). The differences in RNA expression were analyzed.

### RMRP RNA

We created RMRP RNA using pBApo-CMV plasmids (Takara Bio Inc., Shiga, Japan). The RMRP sequence was inserted downstream of the T7 promotor sequence. RMRP RNA was synthesized using a MEGAscript kit (Thermo Fisher Scientific). Huh7 and HLE cells were transfected with 50 pmol/L RMRP RNA using RNAiMAX (Thermo Fisher Scientific). Twenty-four hours after transfection, RNA and protein were extracted from these cells.

### Northern blotting

For Northern blotting, 1 μg RNA was mixed with 3 volumes of formaldehyde loading dye. Samples were applied to lanes of 2% agarose gels, run at 5 V/cm, and transferred onto positively charged nylon membrane (Thermo Fisher Scientific). Membranes were treated by crosslinking, and the RNA was immediately prehybridized. We created a digoxigenin (DIG)-labeled RNA probe (5′-DIG-UGU AGC CUA GGA UAC AG GCC-3′), and after prehybridization, membranes were hybridized overnight with this probe. We then incubated the membranes with an anti-DIG antibody, and signals were detected with CDP-STAR solution (Thermo Fisher Scientific). Signals were visualized with an ImageQuant LAS 4000 system (GE Healthcare, Charles Coffin, NY).

### Western blotting

Protein (20 μg) was applied to lanes of 4% to 12% Bis–Tris Gels (Thermo Fisher Scientific), separated by electrophoresis, and transferred onto Immobilon-P membranes (Millipore, Bedford, MA). Membranes were blocked with albumin and then incubated with the relevant antibodies: anti-PERK (product number: 3192), anti-eIF2α (5324), anti-phospho eIF2α (3398), anti-CCAAT/enhancer-binding protein homologous protein (CHOP) (5554), Bcl-2 (15071), cleaved caspase 3 (9664) (Cell Signaling, Danvers, MA), and anti-actin (1501) (Merck, Darmstadt, Germany) overnight at 4 °C. We used secondary antibodies from kits from GE Healthcare. Bands were labeled using the ECL Prime Kit (GE Healthcare) and visualized with ImageQuant LAS 4000 (GE Healthcare). The density of the bands was quantified by normalization to β-actin using Image J Software (National Institutes of Health, Bethesda, MD, USA).

### Apoptosis assay

Huh7 and HLE cells were transfected with 50 pmol/L control siRNA or RMRP siRNA. The cells were alternatively treated with 1 μg/mL tunicamycin (CAYMAN) dissolved in DMSO or an equivalent volume of DMSO alone and cultured for 24 h. The attached cells were collected and assayed to evaluate apoptosis with annexin V-PE and 7-amino-actinomycin D (Tonbo Biosciences, San Diego, CA). To visualize stained cells, we used a FACSCalibur system (Becton Dickinson, Franklin Lakes, NJ). The data were analyzed with FlowJo software (TreeStar Corporation, Ashland, OR). A Mebstain apoptosis TUNEL kit II (MBL, Nagoya, Japan) was used to detect oligonucleosomal DNA breaks in Huh7 and HLE cells.

### Caspase assay

Huh7 and HLE cells were seeded in 96-well plates. After transfection with control siRNA or RMRP siRNA, Caspase Glo 3/7, Caspase Glo 8, or Caspase 9 Glo reagent (Promega, Southampton, UK) was added to the attached cells, which were cultured for 1 h. Caspase activity was analyzed with a luminometer (GloMax-96 microplate luminometer, Promega).

### Patients and HCC specimens

We obtained HCC samples from patients who underwent surgery at our hospital between 2007 and 2018. The clinical features of the 32 specimens we examined are detailed in Table 1. Rapidly after resection, specimen samples were incubated with RNA later (QIAGEN) overnight at 4 °C and were then frozen and stored at − 80 °C. Remaining samples were fresh frozen and stored at − 80 °C until they were used for protein extraction.

### Ethics statement

The experimental procedures were conducted in accordance with the approved guidelines. The study used clinical samples was approved by the Institutional Review Board (approval ID: 1411010) and was performed in accordance with the ethical principles of the Declaration of Helsinki. Written informed consent was obtained from all patients.

### Statistical analysis

Statistical analyses were performed using JMP version 13.00 (SAS Institute, Cary, NC). Quantitative variables were expressed as means with standard deviations. Statistical differences were determined using the Student’s t-test or a one-way analysis of variance. Correlation coefficients were calculated by Pearson’s correlation coefficient. Differences were considered significant at p < 0.05.

## Supplementary Information

Supplementary Information.

## Data Availability

The datasets generated during and/or analyzed during the current study are available from the corresponding author on reasonable request.
