# Supplementary material for: The long noncoding RNA of RMRP is downregulated by PERK, which induces apoptosis in hepatocellular carcinoma cells
Source: Sci Rep. 2021 Apr 12;11:7926. doi: 10.1038/s41598-021-86592-6 (PMC8041825; doi:10.1038/s41598-021-86592-6)
Supplement: Supplementary file 1 — Supplementary Information. [file 41598_2021_86592_MOESM1_ESM.docx]

**Supplementary Information**

**The long noncoding RNA of RMRP is downregulated by PERK, which induces apoptosis in hepatocellular carcinoma cells**

Atsushi Yukimoto, Takao Watanabe, Kotaro Sunago, Yoshiko Nakamura, Takaaki Tanaka, Yohei Koizumi, Osamu Yoshida, Yoshio Tokumoto, Masashi Hirooka, Masanori Abe, Yoichi Hiasa*

**Supplemental Table 1.** RNA expression differences between cells transfected with control siRNA and PERK siRNA

| Reference | Gene Symbol | log (PERK siRNA  /control siRNA) | *P -*Value |
| --- | --- | --- | --- |
| NR_003051.3 | RMRP | 7.424 | 3.99E-06 |
| NR_110453.1 | CRNDE | 7.162 | 1.72E-05 |
| NM_001001390.1 | CD44 | 6.843 | 8.45E-05 |
| NM_001252093.1 | MRAS | 6.843 | 8.45E-05 |
| NM_002522.3 | NPTX1 | 6.843 | 8.45E-05 |
| NM_004558.3 | - | 6.843 | 8.45E-05 |
| NR_073580.1 | TYSND1 | 6.843 | 8.45E-05 |
| NM_001145310.3 | LRTOMT | 6.652 | 1.98E-04 |
| NM_001256711.1 | ANAPC10 | 6.652 | 1.98E-04 |
| NM_001288739.1 | DNM1 | 6.652 | 1.98E-04 |
| NM_021620.3 | PRDM13 | 6.652 | 1.98E-04 |
| NR_110141.1 | LOC101927870 | 6.547 | 3.09E-04 |
| NM_001318783.1 | LYRM4 | 6.433 | 4.87E-04 |
| NM_001323274.1 | RANBP3L | 6.433 | 4.87E-04 |
| NM_016229.4 | CYB5R2 | 6.433 | 4.87E-04 |
| NM_018363.3 | RNLS | 6.433 | 4.87E-04 |
| NM_018899.5 | PCDHAC2 | 6.433 | 4.87E-04 |
| NR_024347.2 | LINC00304 | 6.433 | 4.87E-04 |
| NR_046853.2 | HM13-AS1 | 6.433 | 4.87E-04 |
| NR_125750.1 | TBX2-AS1 | 6.433 | 4.87E-04 |
| NR_135484.1 | SLC25A35 | 6.433 | 4.87E-04 |
| NR_136734.1 | RNF217 | 6.433 | 4.87E-04 |
| NM_001168338.1 | PLG | 6.308 | 7.77E-04 |
| NM_001282870.1 | RHD | 6.308 | 7.77E-04 |
| NM_001714.2 | - | 6.308 | 7.77E-04 |
| NM_004797.3 | ADIPOQ | 6.308 | 7.77E-04 |
| NM_207491.2 | CCSER1 | 6.308 | 7.77E-04 |
| NR_104635.1 | LOC101926960 | 6.308 | 7.77E-04 |

**
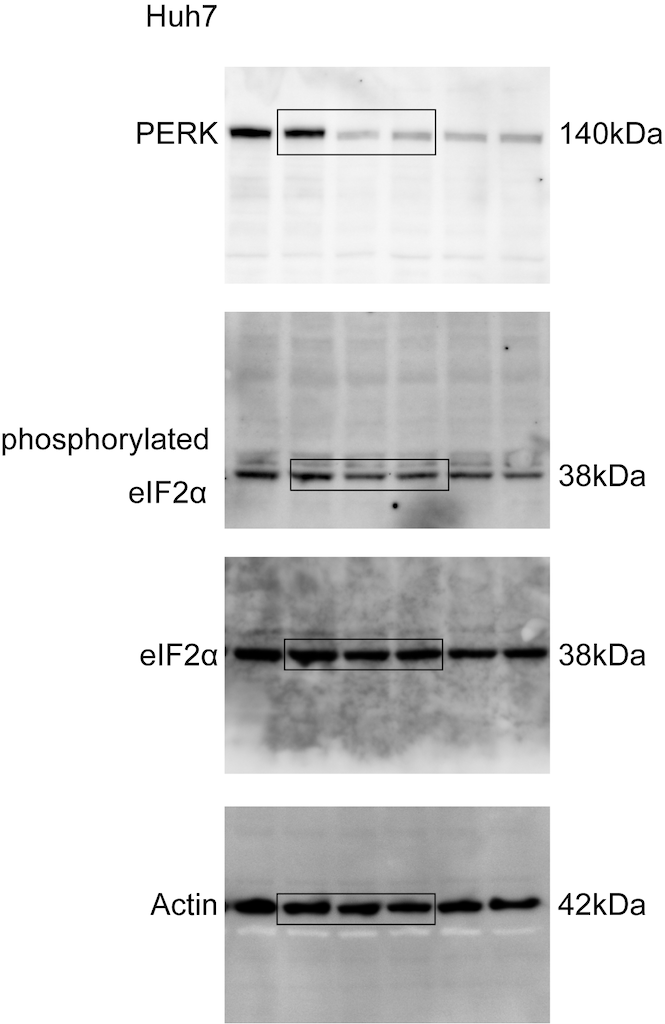
**

**
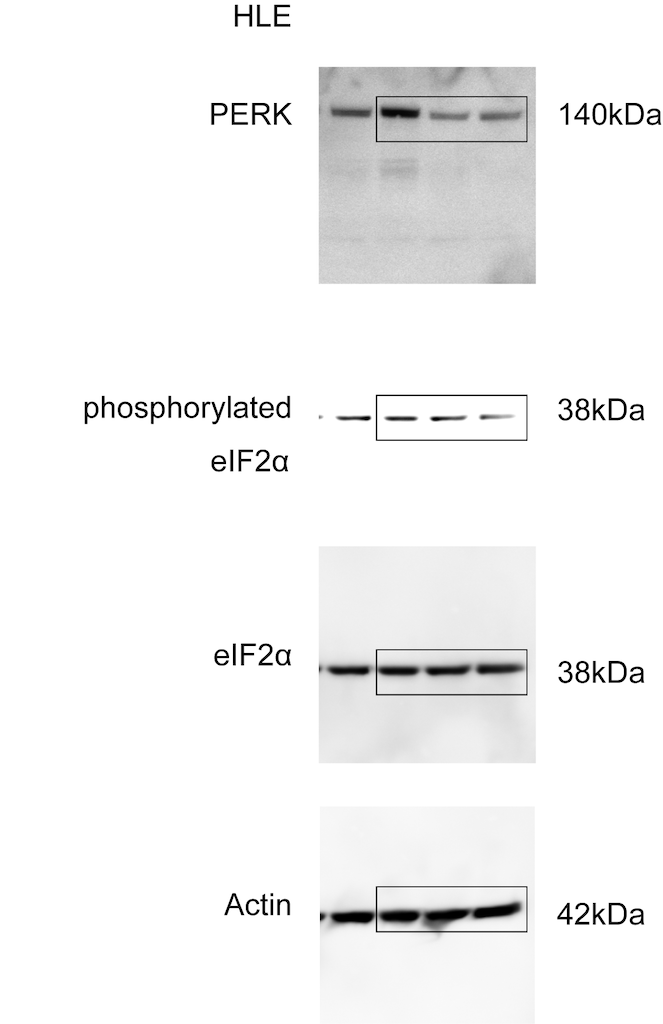
**

**Supplementary Fig. S1.** Original blots for Figure 1B.


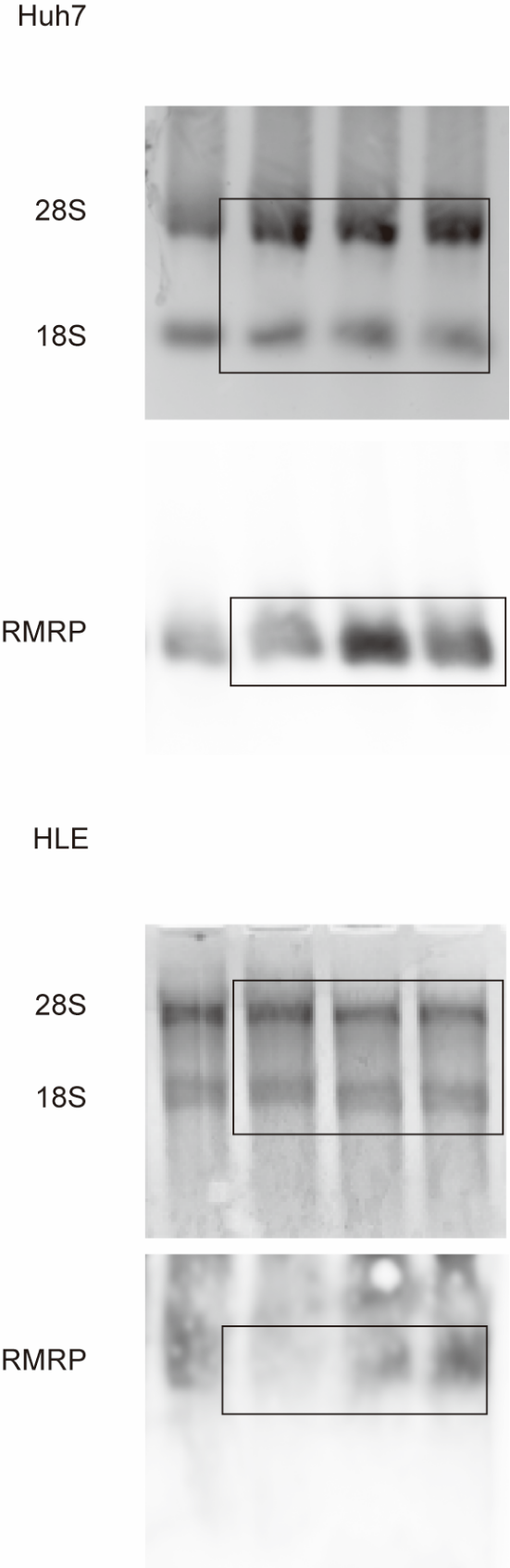


**Supplementary Fig. S2.** Original blots for Figure 1D.


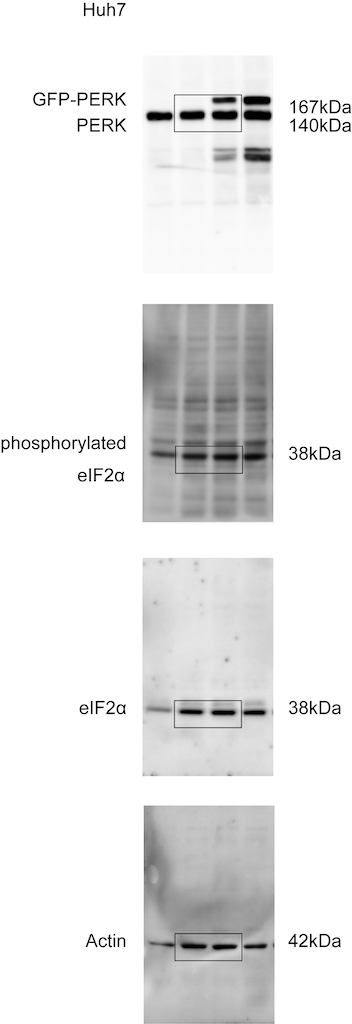


**
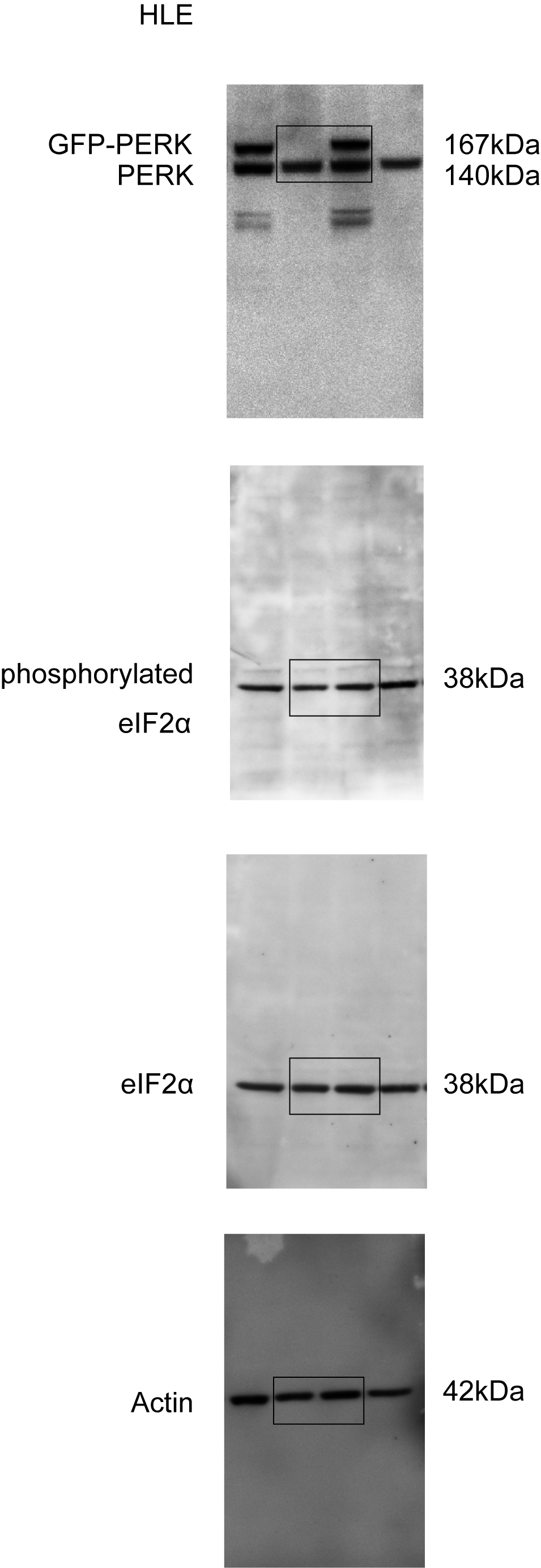
**

**Supplementary Fig. S3.** Original blots for Figure 2B.


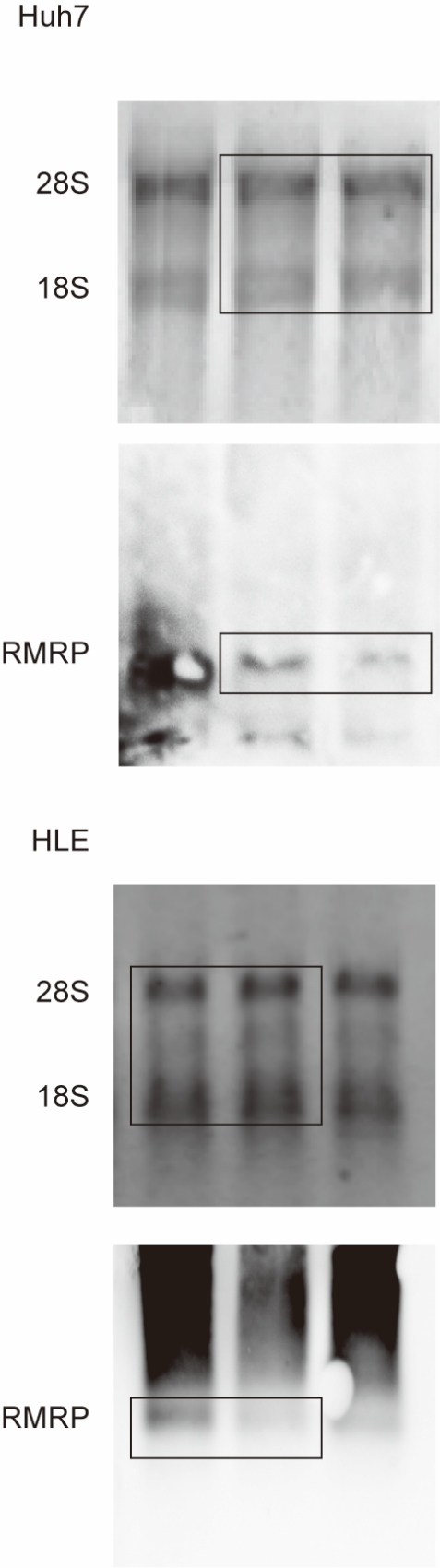


**Supplementary Fig. S4.** Original blots for Figure 2D.


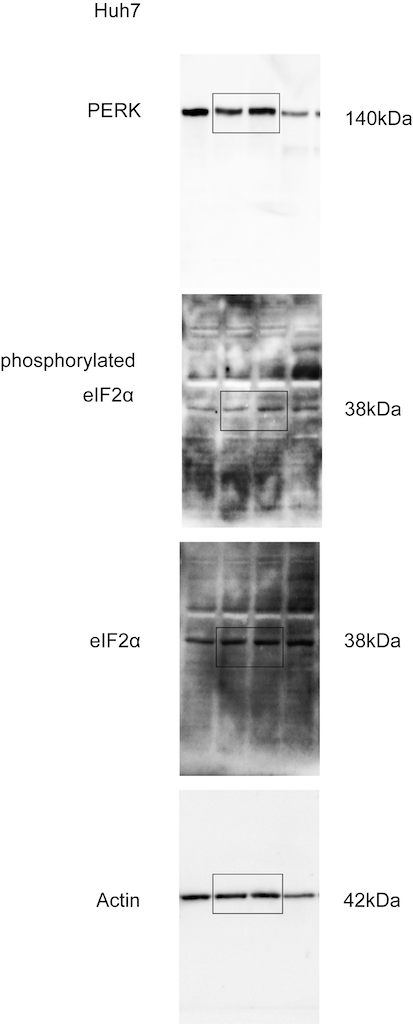


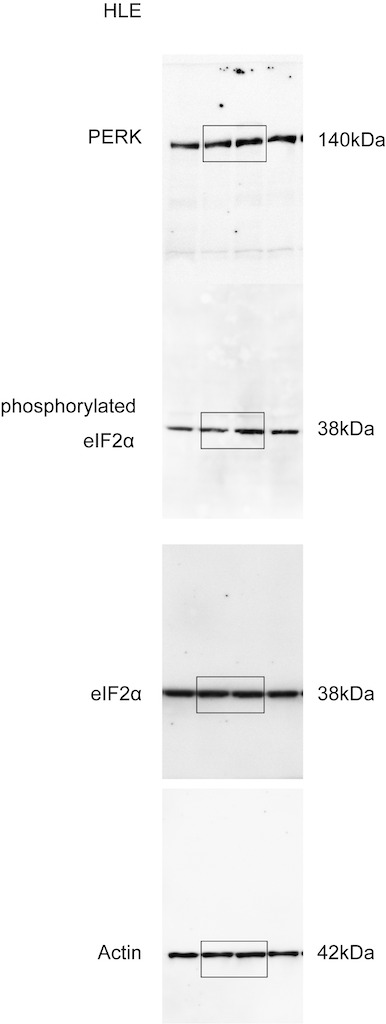


**Supplementary Fig. S5.** Original blots for Figure 3B.


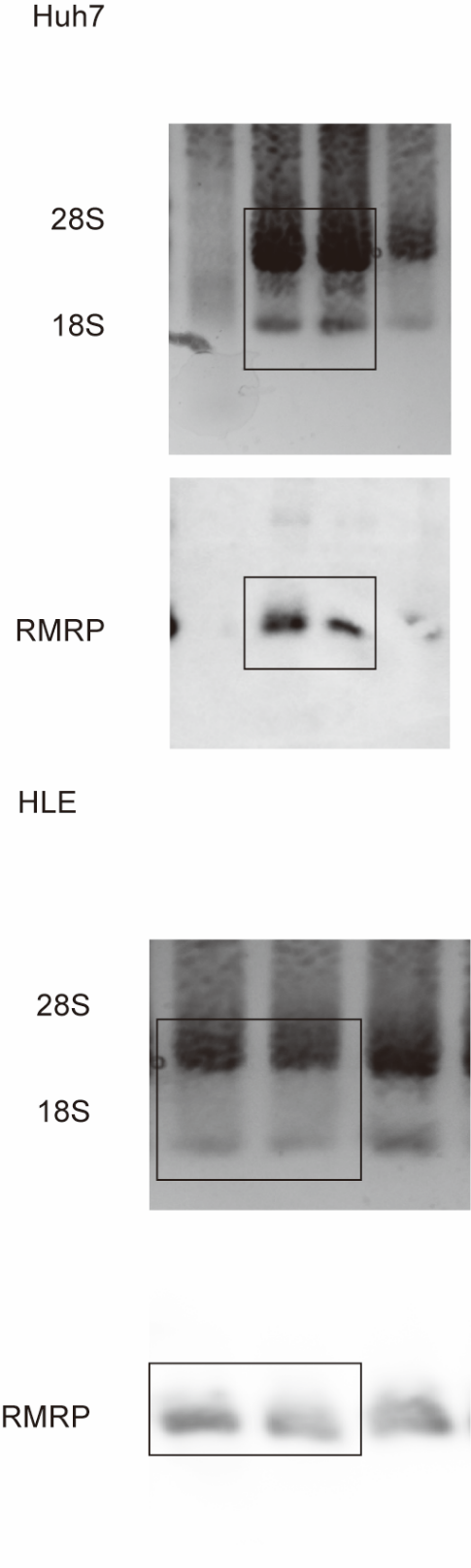


**Supplementary Fig. S6.** Original blots for Figure 3D.


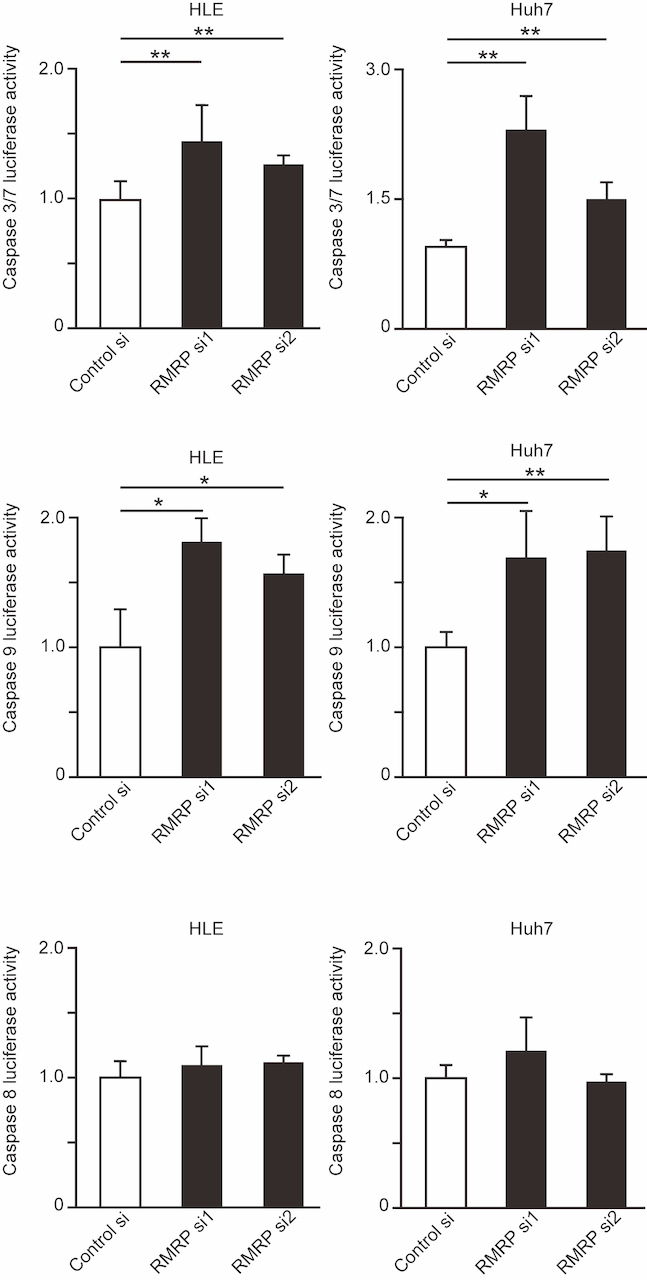


**Supplementary Fig. S7.** **Silencing RMRP leads to activation of caspases.**

Huh7 and HLE cells were seeded in a 96-well flat-bottomed plate, cultured at 37 °C for 24 h, and transfected with control siRNA or RMRP siRNA. Twenty-four hours after treatment, Caspase Glo 3/7, Caspase Glo 8, or Caspase 9 Glo reagent (Promega, Southampton, UK) was added. Caspase activities were analyzed by luminometer. Caspase 3/7 and Caspase9 activities were upregulated by RMRP downregulation. Caspase 8 activity was not changed. Mean ± SEM of six replicates. ******p < 0.01 and *****p < 0.05 the control siRNA group with the Student’s t-test.

**
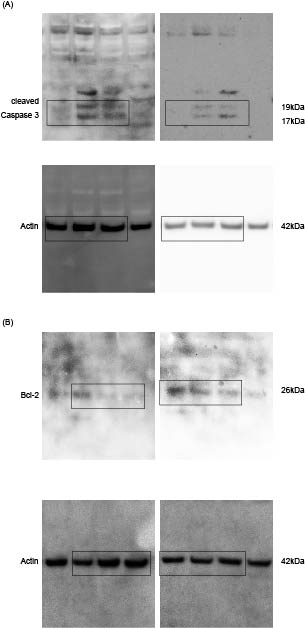
**

**Supplementary Fig. S8.** The original blots for Fig. 5B (A) and Fig. 5C (B).

**
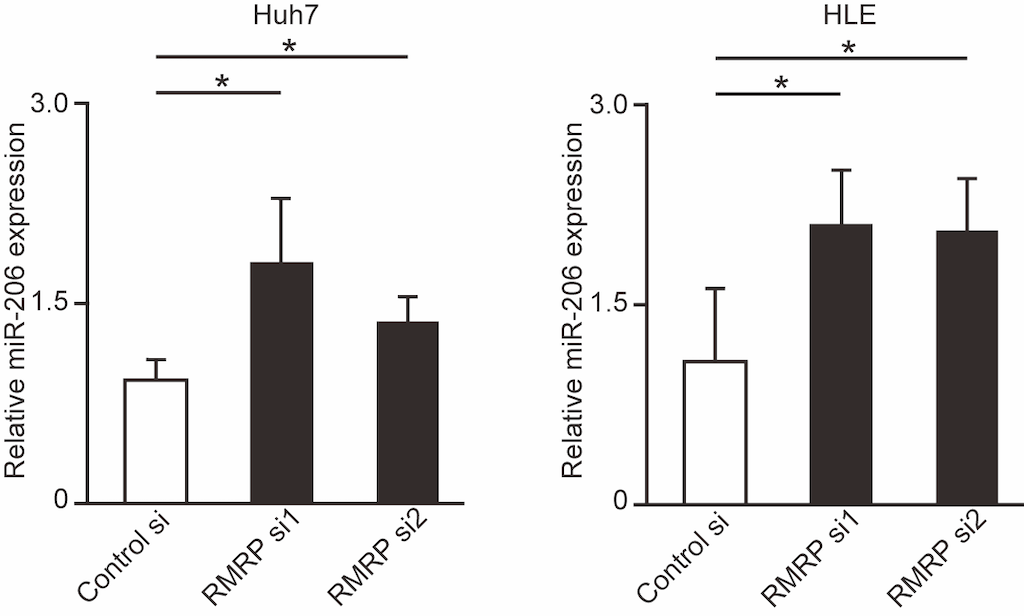
**


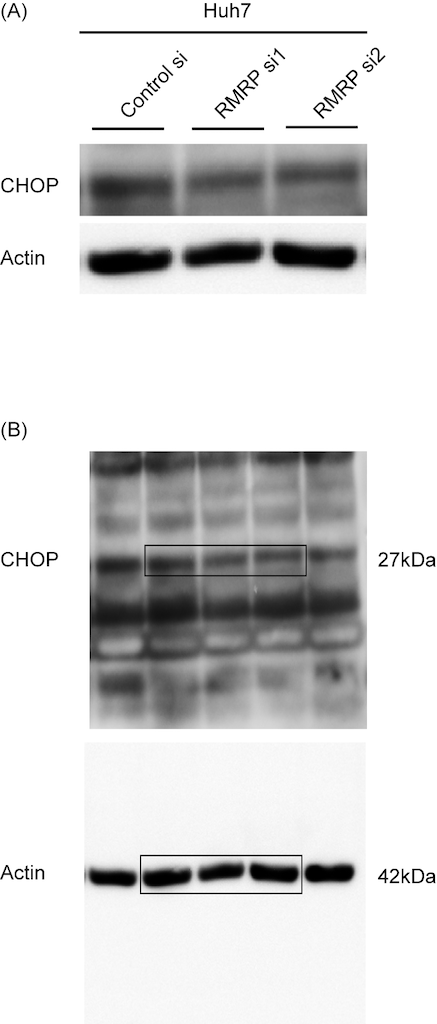


**Supplementary Fig. S9.** **Comparison of CHOP expression between cells transfected with control siRNA and RMRP siRNA.**

Huh7 cells were transfected with control siRNA or RMRP siRNA for 24 h. After transfection, protein was extracted, and levels of CHOP were determined with Western blotting. CHOP expression was not changed between control siRNA group and RMRP siRNA group (A). The original blots for Fig. S10A (B).
